# Age-related differences in dual task walking: a cross sectional study

**DOI:** 10.1186/1743-0003-5-29

**Published:** 2008-11-14

**Authors:** Andrew W Priest, Kathleen B Salamon, John H Hollman

**Affiliations:** 1 Physical Therapy Department, Clarke College, Dubuque, Iowa, USA; 2 Department of Physical Therapy, University of the Pacific, Stockton, CA, USA; 3 Program in Physical Therapy, Mayo Clinic, Rochester, MN, USA

## Abstract

**Background:**

Variability in stride velocity during walking characterizes gait instability and predicts falling in older individuals. Walking while executing a cognitive task is also associated with increased risk of falling, particularly in older adults. Variability in stride velocity, particularly during dual task walking conditions, may differ between younger and older individuals. The purpose of this study was to examine whether gait velocity and variability in stride velocity differ between older community-dwelling women and younger women during dual task walking.

**Methods:**

Twenty-three older (80 ± 9 years) and 19 younger (23 ± 2 years) women walked under each of two conditions: (1) walking at a self-selected velocity and (2) walking at a self-selected velocity while incrementally counting backwards. Gait velocity and variability in stride velocity were measured with GAITRite^® ^instrumentation.

**Results:**

Gait velocity decreased and variability in stride variability increased, in both groups, during dual task walking. The relative reduction in gait velocity and the magnitude of variability in stride velocity were greater in the older subjects than younger subjects.

**Conclusion:**

The gait changes observed in dual task walking characterize reduced gait stability and indicate that cognitively demanding tasks during walking have a destabilizing effect on gait that may place older persons at greater risk of falls.

## Background

Postural stability is classically defined as the ability to control displacements of one's center of mass (COM) relative to one's base of support (BOS) [1]. Walking is a dynamic task whereby the COM and BOS are constantly changing; hence, gait stability can be defined as the ability to control displacements of one's COM in relationship to a constantly changing BOS. Measuring gait stability as defined, however, requires sophisticated laboratory instrumentation that is not entirely conducive to clinical assessment purposes. Rather than measuring COM and BOS relationships during walking, investigators have recognized that stride-to-stride variability in gait predicts falls in older persons and distinguishes those who are at increased falls risk [2,3]. As such, increased stride variability can serve as a marker of gait instability.

Gait instability has been observed in many older adults, even in absence of pathology [4]. In individuals with neurologic pathology, deficits in the central nervous system's ability to coordinate motor outputs are largely responsible for gait instability [5]. It is not entirely clear why gait instability occurs in older individuals who do not have apparent neurologic pathology. The reasons are most likely multifactorial, including deficits in physiologic function such as impaired joint range of motion and muscle performance and deficits in neuropsychological or cognitive status that may exacerbate the effects of impaired physiologic capacity [6-8]. Several studies have examined age-related changes in gait stability over the past decade [3,4,9-13]. Among the findings, initial investigations suggest that increased stride variability may be a more powerful predictor of falling than any of the static measures of balance [3].

Walking has long been considered an automatic or reflex controlled task requiring motor responses to sensory stimuli, but requiring minimal cognitive resources. Given that gait changes are observed in older adults in absence of identified pathology [3,11,13], however, investigators have begun studying the influence of cognitive effects on gait stability using dual task paradigms whereby subjects perform a cognitive task while walking [9,14-16]. Lundin-Olsson, et al. [16], for example, suggested that many falls in balance-impaired older individuals do not typically occur during normal walking conditions, but rather when they are walking and simultaneously performing a secondary task such as talking.

The influence of cognitive activity on gait has been studied in patient populations and results consistently show that persons with neurologic pathology walk with decreased gait velocity and increased gait variability in dual task conditions [17-20]. Studies on the effects of cognitive activity on gait stability in the otherwise well-elderly, however, have had mixed results. Yogev et al. [20] and Springer et al. [21] reported that gait velocity decreased in older healthy subjects during dual task walking but that stride-to-stride variability did not differ between normal and dual task walking conditions. Both studies concluded that the regulation of gait variability does not require attentional capacities in healthy older persons. Alternatively, additional studies have provided evidence that increases in stride variability in addition to decreases in gait velocity occur in healthy older adults who perform a cognitive task while walking [9,10,15,22]. Methodological differences between the studies may account for the contrasting results. Yogev et al. and Springer et al., for example, measured swing time variability over a relatively large number of strides (40–50 strides). Other studies [9,10,15,22], in contrast, measured variability in stride velocity but over fewer strides. Furthermore, Dubost et al. [23] reported that attention-demanding tasks affect stride time variability, independent of changes in velocity, but not stride length variability. It is therefore possible that the variation in gait parameters measured led to the different outcomes among the studies. Perhaps variability in stride velocity, spatial stance and swing parameters or temporal stance and swing parameters reflect different aspects of gait stability. Variability in stride velocity, for example, is a stronger predictor of falls in older adults than other gait parameters [3] and therefore may be more relevant as marker of gait instability than swing time variability. On the other hand, variability in stride velocity measured over relatively few strides (e.g., 11–20 strides reported by Hollman et al. [10]) may be less reliable than the same measure quantified over a greater number of strides.

Given the limited number of strides analyzed in previous studies [9,10,15,22], but recognizing the potential value of measuring variability in stride velocity as it pertains to falls risk in older people [3], assessing whether there are age-related differences in variability in stride velocity during dual task walking over a greater number of strides warrants further investigation. The purpose of this cross-sectional study was to investigate whether variability in stride velocity increases in well-elderly subjects during dual task walking, specifically when compared with younger adults, over a greater number of strides than previous studies have considered. We hypothesized that variability in stride velocity would increase in older subjects during dual task walking and that variability in stride velocity would be greater in older subjects than younger subjects.

## Methods

### Participants

In a pilot study [15] conducted preliminary to the present study, variability in stride velocity in older subjects increased from a coefficient of variation (CV) of 4.9% CV in a normal walking condition to 16.4% CV in a dual task walking condition. To detect a comparable difference in the magnitude of variability in stride velocity of 10% CV, with standard deviations of 7% CV, at *α *= 0.05 and at a statistical power of 0.90, minimally 10 subjects per group were required to participate. For this study we recruited 19 younger and 23 older subjects, a conservative number of subjects estimated to detect a 10-point change in variability in stride velocity at a statistical power of 0.90 or to detect a 5-point change in variability in stride velocity at a statistical power of 0.80. The power analysis was conducted with WINPEPI software [24]. Demographic data are presented in Table 1.

**Table 1:** Demographic Data (mean ± SD)

**Group**	**Age (years)**	**Height (cm)**	**Mass (kg)**
Younger Group (n = 19)	22.7 ± 1.1	167.3 ± 7.8	72.9 ± 12.6

Older Group (n = 23)	79.8 ± 8.6	154.4 ± 8.5	70.2 ± 13.2

Each participant in the older subject group was a self-reported healthy, community-dwelling resident of Mount St. Francis or Mount Carmel, residence facilities for active and retired women who are members of religious orders, in Dubuque, IA, USA. Exclusion criteria consisted of chronic or acute musculoskeletal or neuromuscular pathology that restricted independent walking, a self-reported history of falling, and dependence on an assistive device (e.g., cane or walker) for independent walking. Participants in the younger subject group were volunteers from the student population at Clarke College (Dubuque, IA). All participants provided informed consent. The Clarke College institutional review board approved the study.

### Instrumentation

Data were collected with GAITRite^® ^instrumentation (CIR Systems Inc., Clifton, NJ). The GAITRite^® ^system consists of a 3.66 meter rubberized digital walkway with software for data acquisition and processing. Over 13,000 pressure sensors are embedded within the walkway. As subjects walk across the mat, sensors are activated under pressure at footfall then deactivated at toe-off, enabling spatial and temporal gait data to be collected. Data are sampled at a frequency of 80 Hz, then processed and stored on an IBM compatible computer using GAITRite^® ^Gold software. GAITRite^® ^instrumentation has been reported to have high reliability (ICCs ≥ 0.85) and high concurrent validity when compared with video-based motion analysis systems (ICCs ≥ 0.93) for spatial and temporal parameters of gait such as velocity, cadence, and stride length [25,26].

### Procedures

For the normal walking trials, subjects were instructed to walk at self-selected speeds across the walkway. Under the dual task walking condition, subjects were instructed similarly but in addition they verbally counted backward from 100, subtracting in increments of 3, 4, or 6. The cognitive task we incorporated was similar to the backward-counting-by-3 task used in previous studies [27,28] to manipulate the attention demands of subjects during a motor task. Additionally, to reduce potential practice or learning effects from trial to trial, a different integer was used across successive trials. The order in which each integer (3, 4, or 6) was assigned for each trial in the dual task condition was selected randomly by the investigator. Since we were interested in the potential effects of the cognitive task on changes in gait dynamics and gait instability, and were not necessarily concerned with subjects' performance on the cognitive task itself, we did not evaluate performance on the cognitive task. One investigator walked beside the elderly subjects and adjacent to the walkway during the dual task condition to provide support if a loss of balance occurred. Data from such trials were not included in the analysis, and subjects were asked to repeat the trial.

Subjects initiated each walking trial one meter in front of the walkway, ambulated over the walkway, and terminated the trial one meter beyond the walkway to reduce potential acceleration and deceleration effects of gait initiation and termination on the instrumented walkway. Each walking trial therefore occurred over a distance exceeding 5.5 meters. In the dual task condition subjects started counting backwards as they initiated their walking trials and continued the task until they terminated the trial. Ten walking trials under each condition were recorded for each subject.

The length of the walkway allowed us to collect between three and eight strides during any individual trial, depending on a subject's stride length. While step lengths can vary between right and left sides during walking, a stride is composed of one right step and one left step (or conversely, one left step followed by one right step) and as a result little variation occurs in stride length between right and left sides. We therefore collapsed right and left strides across each of the trials within a walking condition for our data analysis. We collected an average of 57 strides (SD = 20 strides) from subjects in the older age group and 30 strides (SD = 5 strides) from subjects in the younger age group.

Gait velocity (cm/s) was measured directly from the footfalls recorded with the GAITRite^® ^instrumentation. The velocity of individual strides was also recorded with the GAITRite^® ^instrumentation. Stride velocity (cm/s) was calculated as stride length divided by stride time. Stride length is defined as the linear distance (in cm) between successive heel contacts of the same foot. Stride time is defined as the duration (in seconds) over which one stride occurs.

### Data Analysis

We operationally defined gait instability as variability in stride velocity. We quantified variability in stride velocity as the percentage coefficient of variation (CV) across multiple strides collected from ten walking trials. CV is determined by the equation,

$$CV=\left(SD/\bar{X}\right)\times 100$$

where SD = standard deviation and $\bar{X}$ = mean. The CV is a measure of relative variation most meaningful when comparing the variability of distributions, such as that obtained from two groups of subjects. In addition to variability in stride velocity, we also measured average gait velocity in both subject groups. Gait velocity, while less powerful an indicator of gait instability than stride variability [3], is commonly used to characterize dynamic gait kinematics.

Descriptive data (mean ± SD) across walking conditions were calculated. Two 2 × 2 mixed model analyses of variance (ANOVAs) having one between-subjects factor (group: younger and older subjects) and one within-subjects factor (condition: normal and dual task walking) were conducted to analyze differences in mean gait velocity and variability in stride velocity (*α *= 0.05). Post hoc t-tests with the Bonferroni-adjusted *α *were conducted when necessary to identify the comparisons that were statistically significant.

## Results

**Figure 1:**
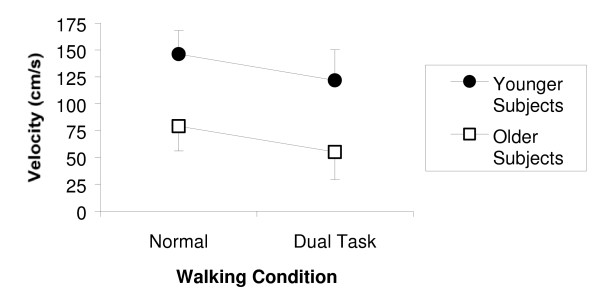
**Gait Velocity**. Gait velocity in the normal and dual task walking conditions (error bars represent one standard deviation). The difference in gait velocity between the normal and dual task walking conditions is statistically significant [F(1,40) = 61.713, p < 0.001]. The difference in gait velocity between older and younger subjects is statistically significant [F(1,40) = 90.247, p < 0.001].

Gait velocity data are presented in Figure 1. Older subjects walked more slowly than younger subjects during both walking conditions [F(1,40) = 90.25, p < 0.001]. Each subject, whether younger or older, walked more slowly in the dual task walking condition than in the normal walking condition [F(1,40) = 61.71, p < 0.001]. On average, gait velocity decreased by 18% in the younger group and gait velocity decreased by 30% in the older group [t(40) = 2.118, p = 0.040].

**Figure 2:**
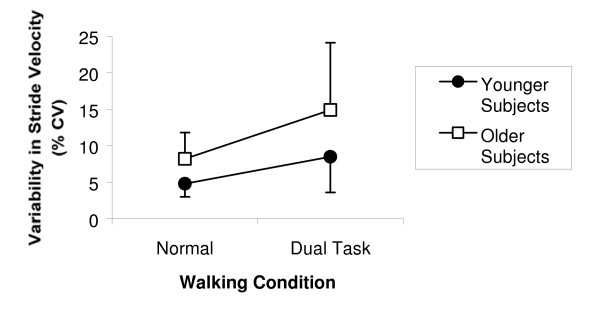
**Variability in stride velocity**. Variability in stride velocity in the normal and dual task walking conditions, as quantified with the coefficient of variation (error bars represent one standard deviation). The difference in variability in stride velocity between the normal and dual task walking conditions is statistically significant [F(1,40) = 20.281, p < 0.001]. The difference in variability in stride velocity between older and younger subjects is statistically significant [F(1,40) = 13.232, p = 0.001].

**Figure 3:**
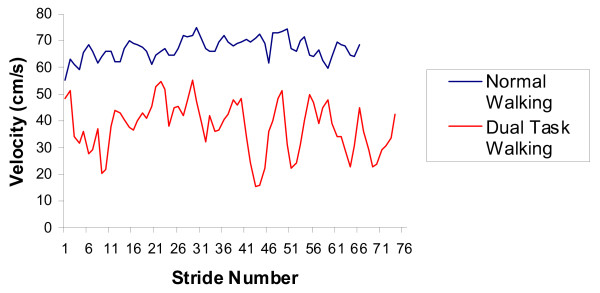
**Characteristic example of stride velocity**. Characteristic example of stride velocity in an 87 year old woman. Mean velocity in the normal walking condition is 67.1 cm/s and variability in stride velocity, as quantified with the coefficient of variation (CV), is 5.9% CV. Mean velocity in the dual task walking condition is 37.6 cm/s and stride-to-stride variability in velocity is 25.3% CV.

The effects of cognitive activity on stride variability are illustrated in Figures 2 and 3. Figure 3 presents a characteristic example of the effect of dual tasking on both gait velocity and variability in stride velocity. Overall, older subjects walked with greater variability in stride velocity than younger subjects during both walking conditions [F(1.40) = 13.23, p = 0.001]. Both groups of subjects walked with greater variability in stride velocity in the dual task walking condition than in the normal walking condition [F(1,40 = 20.28, p < 0.001]. In younger subjects, relatively little variability in stride velocity was observed in the normal walking condition (4.8 ± 1.8% CV); variability in stride velocity increased in the dual task walking condition (8.5 ± 4.9% CV). The difference in variability in stride velocity between conditions in younger subjects was statistically significant [t(18) = 3.608, p = 0.002]. Similarly, older subjects walked with less variability in stride velocity in the normal walking condition (8.2 ± 3.6% CV) than in the dual task walking condition (14.9 ± 9.2% CV); the difference in variability in stride velocity between conditions was statistically significant [t(22) = 3.475, p = 0.002]. The greatest variability in stride velocity observed in the study occurred among older subjects in the dual task walking condition [t(40) = 2.714, p = 0.010].

## Discussion

Similar to the results of previous studies [9,10,15,22], this study provides evidence that cognitive activity during walking reduces gait velocity and increases variability in stride velocity in well-elderly women. The magnitude of reduction in gait velocity observed among older subjects in dual task walking in the present study, approximately 24.0 cm/s, is comparable to the 24.5 cm/s reduction reported by Hollman et al. [10]. The magnitude of increase in variability in stride velocity during dual task walking, approximately 6.7% CV, is comparable to the 6.9% CV magnitude of change reported by Beauchet et al. [9].

A strength of the present study is that the number of strides analyzed per subject increased two- to several-fold compared to previous studies measuring similar gait parameters [9,10,15,22]. While the number of strides required to measure variability in stride velocity reliably is not known, previous studies indicate that limited stride numbers may influence the measure's reliability. Besser et al. [29], for example, reported that 5 to 8 strides are necessary for 90% of individuals tested with GAITRite^® ^instrumentation to have reliable mean estimates of spatiotemporal gait parameters including velocity, stride and step length, and step and single support time. Other parameters, including base of support width and double support time, required greater than 10 strides to yield reliable data. Additional research indicated that while measurement of spatiotemporal gait parameters including velocity and cadence is highly reliable with GAITRite^® ^instrumentation when subjects perform 3 trials per test [30], measurement of variability in stride velocity is less reliable with a similar number of trials and therefore may require that a greater number of strides be analyzed [31]. Based on such studies, it is evident that analyzing greater numbers of strides produces more reliable gait data when the parameters being measured are inherently more variable.

While we cannot ascertain precise reliability coefficients of the data measured in the present study, we can apply the Spearman-Brown prophecy formula to estimate reliability of variability in stride velocity data. The Spearman-Brown prophecy formula is denoted as [32]

$$\rho^{*}=\frac{N\rho_{xx'}}{1+\left(N-1\right)\rho_{xx'}}$$

where ρ* is the projected reliability coefficient, *N *represents the additional sets of strides analyzed, and *ρ*_*xx*' _is the known reliability coefficient. Assuming that the test-retest reliability coefficient for variability in stride velocity during normal walking is 0.66 based on data collected over 13 strides [31], for example, application of the Spearman-Brown prophecy formula indicates that the projected reliability of variability in stride velocity increases to approximately 0.90 when data are collected over 57 strides, a factor 4.4 times greater than the number of strides collected in the cited study. Stride variability data obtained in the present study are therefore projected to be more reliable than results of similar work [9,10,15,22] in which GAITRite^® ^instrumentation has been used to quantify stride variability. Since results in the present study are consistent with results of those studies, confidence is enhanced that the dual tasking effects represent real effects of cognitive demands on gait performance rather than natural variations that may occur in gait.

An increase in variability from one stride to the next, whether the measure reflects variability in step length [33], variability in stride time [4,5,17,19], or variability in stride velocity [3,9,10,15,22], reflects an impaired ability to regulate stride-to-stride variations in gait timing. The increase in stride variability therefore reflects gait instability. We chose to analyze specifically subjects' variability in stride velocity because, of the various gait parameters reflecting gait instability, it appears to be the best predictor of future incidence of falls in elderly individuals [3]. Lundin-Olsson et al. [16] and others [34] have suggested that cognitive activity during walking may increase the risk of falling in older individuals. While this study did not examine risk of falling, results concur that performing a cognitive activity during walking does influence gait instability in older women. The threshold at which variability in stride velocity during dual task walking predicts an elevated risk of falling has not, to our knowledge, been determined. Nevertheless, we believe that the consequence of cognitive activity during walking needs to be considered by clinicians and researchers alike who work with individuals with impaired balance or with those who may be at elevated risk of falling.

Results of the study have several clinical implications. Foremost, a clinician must recognize that attention-demanding tasks have a destabilizing effect on gait, particularly in older individuals. Recognizing the influence of cognitive activity on gait and gait stability, a clinician may instruct older individuals who are at risk of falling to avoid performing cognitive tasks while they are walking. Alternatively, a clinician may recognize the utility of dual tasking and choose to engage the individual in cognitive activities while walking in an effort to improve the person's ability to perform dual tasks in a safe and functional manner. While no large scale studies, to our knowledge, have addressed these issues from a clinical perspective, Maki [3] and Hausdorff et al. [35] suggest that gait stability improves with exercise, although they did not examine the question under dual task walking conditions. Silsupadol et al. [36] provide evidence through case reports that two patients who received balance training under dual task conditions showed benefits maintained over 3 months that were not evident in a patient who trained under a single task balance training program. Whether training under dual task conditions can improve gait or fall risk during dual task walking needs further investigation.

Interpreting results of the present study is limited somewhat by its relatively nonspecific inclusion and exclusion criteria. Each older subject described herself as a community-dwelling, healthy woman without a history of falls and without pathology that restricted independent walking. There are, however, potentially many other factors not addressed in pre-test screening that may have otherwise classified the subjects as being at risk for falling or as being susceptible to gait instability. Examples include cognitive status [37], medication history [38,39], other measures of static or dynamic balance [37] and other measures of physical function [40]. For instance, some of the data in our study suggest that the older subjects we studied may not have been entirely void of fall risk. Reduced gait velocity is not necessarily a predictor of gait instability or a risk factor for falls, but may be a symptom of a person's fear of falling [3]. The older subjects in our study walked more slowly (79 cm/s) than similar healthy, community-dwelling subjects in other studies (approximately 100 cm/s [33,35]) and some, such as the individual represented in Figure 3, may have walked at gait velocities below that typically seen in community ambulators [41] or may have been at risk of falling. Additionally, the study included women only and therefore results should not be generalized to men. Despite the limitations, the study adds to a growing body of evidence [9,10,15,22] that dual tasking influences gait performance and gait stability and that the effect is particularly pronounced in older individuals.

## Conclusion

Gait velocity decreased and variability in stride velocity increased, in both young women and older community-dwelling women, during dual task walking. The relative reduction in gait velocity and increased magnitude of variability, however, were more pronounced in the older participants. Gait variability observed in the dual task walking condition characterizes impaired execution of gait that reflects gait instability and indicates that cognitively challenging tasks performed while walking may place older persons at greater risk of falls.

## List of abbreviations

ANOVA: analysis of variance; BOS: base of support; COM: center of mass; CV: coefficient of variation; ICC: intraclass correlation coefficient; SD: standard deviation

## Competing interests

The authors declare that they have no competing interests.

## Authors' contributions

AWP participated in the design of the study, participated in the data collection, and contributed to the writing of the document. KBS participated in the design of the study, participated in the data collection, and contributed to the writing of the document. JHH participated in the design of the study, conducted the data analysis, and contributed to writing of the document. All authors read and approved the final manuscript.
